# Decrease of Serum Angiotensin Converting Enzyme Levels Upon Telbivudine Treatment for Chronic Hepatitis B Virus Infection and Negative Correlations Between the Enzyme Levels and Estimated Glumerular Filtration Rates

**DOI:** 10.5812/hepatmon.15074

**Published:** 2014-01-31

**Authors:** Kung-Hao Liang, Yi-Cheng Chen, Chao-Wei Hsu, Ming-Ling Chang, Chau-Ting Yeh

**Affiliations:** 1 Liver Research Center, Chang Gung Memorial Hospital, Taipei, Taiwan; 2 Molecular Medicine Research Center, Chang Gung University, Taoyuan, Taiwan

**Keywords:** Hepatitis B, Nucleosides, Antiviral Agents, Kidney

## Abstract

**Background:**

During antiviral therapy for chronic hepatitis B, renal function impairment could be a critical concern when oral nucleot(s)ide analogues were used. Paradoxically, long-term telbivudine treatment was associated with an increase of estimated glomerular filtration rate (eGFR) through unknown mechanisms.

**Objectives:**

We aimed to investigate changes in serum protein abundances associated with renal function in response to antiviral treatments.

**Materials and Methods:**

Primarily, a transcriptomic assay was performed to identify differentially expressed genes in peripheral blood cells caused by the telbivudine treatment. Two genes coding angiotensin converting enzyme (ACE) and complement factor H (CFH) were screened from 14 candidate renal function-related genes. ACE and CFH production were further investigated using enzyme-linked immunoassays.

**Results:**

Verification studies showed no significant change of serum CFH levels, but there was a significant reduction of serum ACE levels by continuous telbivudine treatment for 330.00 ± 0.85 days (34 patients; paired t-test, P = 0.022). Serum HBV DNA and ALT levels also decreased (P = 0.008 and < 0.001, respectively). A significant increase in eGFR was found (33 patients, paired t-test, P = 0.002) at 708.64 ± 31.63 days. Patients’ eGFRs were negatively correlated with serum ACE levels (r = -0.375, P = 0.002) but not with serum HBV DNA and ALT levels (P = 0.241 and 0.088 respectively). Significant decreases of the ACE levels were also observed upon entecavir treatment (20 patients; paired t-test, P = 0.020) at 412.88 ± 36.92 days. No significant correlation was found between serum ACE levels and eGFRs (r = -0.239, P = 0.138) in entecavir-treated patients.

**Conclusions:**

We discovered a consistent reduction of serum ACE levels by two oral antiviral monotherapies, entecavir and telbivudine. Serum ACE levels were negatively correlated with eGFRs in telbivudine treated patients.

## 1. Background

Chronic hepatitis B virus (HBV) infection is a prevalent disease with more than 350 million people affected globally (1). The chronic infection lasts for decades and even through an entire lifetime. It is important to control the HBV-induced liver damage, while at the same time, maintaining functions of other major organs including kidneys. The natural history of chronic hepatitis B represents the everlasting battle between HBV and the host, which comprises four stages: immune tolerance, immune clearance, low replicative and reactivation stages (2-4). Marked and repeated elevations of alanine transaminase (ALT) suggest that the disease has progressed to the immune clearance or reactivation stages. Prolonged ALT flare indicates severe and continuous liver damage which leads to subsequent complications such as liver fibrosis, cirrhosis and hepatocellular carcinoma. Therefore, medical intervention is recommended upon ALT elevation according to international guidelines (2).

Interferon therapy is the first FDA-approved treatment for HBV infected patients, which has recently been replaced by peginterferon treatment with markedly improved efficacy. A series of nucleos(t)ide analogues acting as reverse transcriptase inhibitors, including lamivudine (4), adefovir (5), entecavir (5), telbivudine (6, 7) and tenofovir (6), were developed and approved subsequently. They are oral antiviral drugs designed to inhibit viral replication, and their potent effects of viral load reduction and serum ALT normalization have been well demonstrated. However, various para-antiviral effects such as renal function changes during long-term treatment were also observed. Adeforvir and tenofovir can cause renal toxicity particularly at high doses (2, 5). In contrast, telbivudine (LdT) has been shown to improve renal functions from baseline via an unknown mechanism after continuous treatment for more than 60 weeks (7).

Renal function is often gauged by the estimated glomerular filtration rate (eGFR), whose value above 90 (ml/min/1.73 m2) is considered normal, and between 60 and 90 mild impairment is considered (8). Viral antigens such as core, e- and surface-antigens (HBcAg, HBeAg and HBsAg, respectively) have long been observed to accumulate in kidneys, impairing the renal filtration capability (9-11). As current oral antiviral drugs can only suppress viral replication but cannot eliminate HBsAg (5, 6), renal function improvement is unlikely ascribed to the reduction of HBsAg-derived immune complex in kidneys.

ACE inhibitors have a well-established therapeutic effect in halting the progression of various kidney diseases (12, 13). ACE plays an important role in the renin-angiotensin aldosterone regulatory system, with known downstream effects on systemic vasoconstriction and renal sodium and renal fluid retentions. Other effects of ACE include the inactivation of bradykinin (a vasodilator), and the reduction of N-acetyl-seryl-aspartyl-lysyl-proline (14, 15). Due to the above reasons, suppression of ACE levels by LdT treatment may contribute toward renal function improvement.

## 2. Objectives

In this study, we explored the molecular alterations in response to two commonly prescribed oral antiviral drugs, telbivudine and entecavir. Serum proteins are attractive study targets due to their clinical effects on capillaries, the main components of renal glomeruli. Thus, changes of serum proteins were evaluated to correlate with eGFR.

## 3. Materials and Methods

### 3.1. Study Subjects

This study was conducted under the approval of Institutional Review Board, Chang Gung Memorial Hospital, Taiwan. All study subjects were adult and treated at the Linko Medical Center of Chang Gung Memorial Hospital. All of subjects signed informed consent forms. LdT was administered at a dose of 600 mg per day (6). Entecavir was given at 0.5 mg per day (5).

This study was initially a transcriptomic exploration, followed by clinical verifications of serum proteins. We aimed to explore hepatitis-independent, drug related molecular alterations in the exploratory phase. 4 subgroups of subjects were retrospectively recruited, including inactive carriers (n = 3), patients before treatment (baseline, n = 2), patients receiving LdT treatment for 24-36 weeks (0.5 year, n = 2), and patients receiving LdT treatment for 40-60 weeks (1 year, n = 3). Inactive carriers were characterized by normal serum ALT levels, suggesting that the HBV viruses do not cause severe damage to the host liver (16). The four subgroups were used separately as well as in combination for the exploration. The baseline, 0.5 year and 1 year subgroups were used to check a time-dependent increase or decrease of the gene levels. In the first step of the screening, inactive carrier and baseline subgroups were combined as the “non- treated” group. The 0.5 year and 1 year subgroups were combined as “LdT-treated” subjects. Genes level differences between LdT-treated and non-treated groups were presumably ascribed to the drug effect. These genes were identified as the primary candidates. In the second step of the screening, only differentially expressed genes with large increase or decrease rates from baseline, 0.5 year to 1 year, were considered. In the final step, only renal function related genes were listed.

In the subsequent verification phase, two independent cohorts were retrospectively recruited. They were patients treated either by LdT (n = 34) or entecavir (n = 20). Serum samples at baseline and after treatment were submitted for candidate factor analysis. Clinical variables including age, gender, cirrhosis, body weight, serum AST and ALT levels, bilirubin, albumin, HBV viral load, serum creatinine level, HBeAg and HBsAg were recorded. Values of age, gender and serum creatinine level were used for the calculation of eGFR according to the Modification of Diet in Renal Disease (MDRD) equation (8):

GFR = 186 × (Scr)-1.154 × (age)-0.203 × 0.742 (if the subject is female).

### 3.2. Transcriptomic Explorations

The Affymetrix PrimeViewTM human gene expression platform was used for the transcriptomic exploration (Affymetrix, Santa Clara, CA). Peripheral blood cell samples were stored at -70C and thawed at room temperature for subsequent assays. Cellular RNA was extracted by the use of TRI Reagent RT (Molecular Research Center Inc., Cincinnati, OH). Reverse transcription was then conducted using the T7 oligo (dT) primer to obtain the corresponding cDNAs. An *in vitro* transcription (IVT) with biotinylated ribonucleotide analog was then performed to generate biotin-labeled amplified RNA (aRNA), using GeneChip 3’IVT Express kit (Affymetrix, Santa Clara, CA). The aRNAs were then purified by magnetic beads and fragmented for the subsequent hybridization according to the manufacturer’s protocol. Streptavidin-phycoerythrin, which binds to biotin-labeled aRNA was then used as the fluorescent dye. Fluorescent signal was scanned using the GeneChip Scanner 3000 7G (Affymetrix, Santa Clara, CA) to represent aRNA levels. The signal readings were log2-transformed and then normalized across all the samples using the Robust multiarray analysis (RMA) algorithm before comparison across clinical groups (17). The microarray data can be accessed via the European Bioinformatics Institute ArrayExpress repository (http://www.ebi.ac.uk/arrayexpress/) by the accession number E-MTAB-1457.

### 3.3. ACE and CFH Quantitation by Enzyme-Linked Immunosorbent Assay (ELISA)

Verification was performed by comparing serum protein levels of subjects before and after continuous telbivudine or entecavir treatments. Serum samples were stored at -70°C and thawed at room temperature for subsequent assays. Serum angiotensin converting enzyme (ACE) levels were assayed by Human ACE immunoassay (Quantikine, Minneapolis, MN). Serum complement factor H (CFH) levels were assayed by the ELISA Kit ABIN414509 (antibodies-online Inc., Atlanta, GA). 

### 3.4. Statistical Analysis

Mann-Whitney tests, analysis of variance (ANOVA) and Tukey’s post hoc analysis were employed to compare the clinical parameters of the exploratory study. Two filtering criteria were employed for excluding irrelevant genes from further analysis: (i) no significant difference between LdT-treated and non-treated groups (P > 0.05), or (ii) insufficient time response upon a continuous LdT treatment (∣βg∣ < 0.2). βg was estimated for each gene (g) by a linear regression of log2-transformed and normalized signal readings (log_2 _(yg,t)) with respect to time:

log_2 _(yg,t) = αg + βg × t + εg

Where εg is the error term; t is the time parameter (year). αg (the regression constant) and βg were calculated by the standard least-square methods. Positive and negative values of βg indicate time-dependent increments and decrements of signal readings from baseline. The estimated fold change of signal at one year versus baseline was 2βg. 

Paired statistical tests were employed in the verification phase to see whether a significant alteration of serum proteins could be observed upon treatment. This was performed by a parametric paired t-test. Pearson’s correlations (r) were used to estimate the relationship between serum protein levels and eGFR. Average values were presented as mean ± standard error. 

## 4. Results

### 4.1. ACE RNA Was Monotonically Decreased While CFH Increased Upon LdT Treatment 

**Figure 1.**
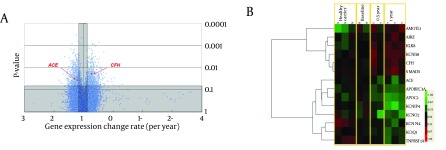
Transcriptomic Alterations by LdT (A) Horizontal axis shows gene level change rate (βg). Vertical axis indicates significance level. Circles represent genes. Shaded area contains genes excluded by the criteria. (B) Levels of 14 kidney-related genes (rows) in 10 subjects (columns). Colours represent the fold change from the geometric mean of the baseline group. Green colours represent decreases from the baseline, while red colours represent increases.

Age, gender, serum HBV DNA level and ALT were not significantly different between LdT-treated and non-treated groups based on the Mann-Whitney test (Table 1). Only the ALT levels of baseline subgroup was significantly different from those of the other three groups (ANOVA P < 0.001, Tukey’s post hoc P ≤ 0.001). A volcano plot showed changes in gene levels per year (βg; the horizontal axis of Figure 1A), and the P values comparing the non-treated and LdT treated groups (the vertical axis of Figure 1A). A total of 709 genes remained after filtering by the two criteria (see methods). Among them, 16 genes were pertinent to kidney functions (Table 2, Figure 1B), and two of them became the focus of our attention: ACE and CFH. ACE was reduced monotonically in response to LdT treatment, while CFH was elevated. Serum ACE and CFH proteins may mediate renal damage or protection through circulation. Thus, the two genes were selected for subsequent verification.

**Table 1.** Basic Clinical Parameters of HBV Carriers in the Exploratory Study

	Non-treated		LdT-treated		Mann-Whitney P ^a^	ANOVA P ^b^
	Inactive Carrier	Baseline	LdT 0.5 year (24-36 weeks)	LdT 1 year (40-60 weeks)		
**Subject Number**	3	2	2	3		
**Age, y**	40.67	41	42	44.33	0.53	0.947
**Gender, %**	Male, 100	Male, 100	Male, 100	Male, 100	1	1
**Serum HBV DNA, ×10** ^**6**^ ** copies ml-1**	0.02	7.85	0	0.15	0.18	0.285
**Serum ALT level, IU L-**1	20	251	35	53.67	0.92	< 0.001

^a^ The non-parametric Mann-Whitney test was used to compare Non-treted vs. LdT-treated groups.

^b^ANOVA test was used to compare the four groups.

**Table 2.** Genes Associated With Renal Functions

Gene Symbol	Gene Name	P value	βg	Estimated Fold Change, 1 year / baseline
**KCNIP4**	Kv channel-interacting protein 4	0.016	-0.543	0.686
**APOC2**	Apolipoprotein C-II	0.010	-0.432	0.741
**KCNQ2**	Potassium voltage-gated channel subfamily KQT member 2	0.020	-0.352	0.784
**APOBEC3A**	Apolipoprotein B MRNA Editing Enzyme, Catalytic Polypeptide-Like 3A	0.007	-0.325	0.798
**PLA2G6**	phospholipase A2, group VI (cytosolic, calcium-independent)	0.034	-0.309	0.807
**PLA2G12A**	phospholipase A2, group XIIA	0.004	-0.257	0.837
**KCNQ1**	Potassium voltage-gated channel subfamily KQT member 1	0.011	-0.254	0.839
**TNFRSF1A**	Tumor necrosis factor receptor superfamily member 1A	0.039	-0.230	0.853
**KCNN4**	Intermediate conductance calcium-activated potassium channel protein 4	0.022	-0.208	0.866
**ACE**	Angiotensin-converting enzyme	0.037	-0.207	0.866
**AIRE**	Autoimmune regulator	0.044	0.219	1.164
**CFH**	Complement factor H	0.019	0.269	1.205
**KLK8**	Kallikrein-8	0.002	0.282	1.216
**AMOTL1**	Angiomotin-like protein 1	0.036	0.311	1.241
**KCNK18**	Potassium channel subfamily K member 18	0.009	0.332	1.258
**SMAD5**	SMAD family member 5	0.040	0.332	1.258

### 4.2. Concentration of ACE Protein but not CFH Was Significantly Reduced Upon Oral Antiviral Treatment

Serum ACE protein concentration was reduced significantly at 330.00 ± 0.85 days of LdT treatment (34 patients, P = 0.022, Table 3). In contrast, serum CFH levels did not show a significant difference in response to treatment (Table 3). Thus, CFH was not investigated further. Serum ACE levels were also reduced at 412.88 ± 36.92 days of entecavir treatment (20 patients, P = 0.020, Table 3). As expected, serum HBV DNA and ALT were both suppressed by the two oral antiviral treatments, showing the effectiveness of the treatment. The proportions of subjects with viral antigens were unchanged after treatments. 

**Table 3.** Clinical Data at Baseline and After Antiviral Treatments ^a^

		Baseline	After Treatment ^b^	P value
**LdT ^c^ Cohort (n = 34)**				
Age, Mean ± SD, y		46.68 ± 1.87		
Gender, Male (%)		29 (85.3)		
Cirrhosis, (%)		13 (38.24)		
Body weight, Mean ± SD		67.63 ± 1.92		
HBeAg ^c^ positive, (%)		9 (26.5)	9 (26.5)	
HBsAg positive, (%)		34 (100)	34 (100)	
Serum ALT ^c^, Mean ± SD, IU L-1		184.15 ± 31.79	43.91 ± 11.73	< 0.001
Serum AST ^c^, Mean ± SD, IU L-1		104.53 ± 16.30	44.71 ± 8.19	0.002
Serum HBV ^c^, Mean ± SD, DNA ×10^6^ copies mL-1		180.63 ± 58.24	11.73 ± 11.09	0.008
Serum ACE ^c^, Mean ± SD, ng mL-1		284.98 ± 49.12	253.58 ± 41.38	0.022
Serum CFH ^c^, Mean ± SD, ug mL-1		1101.32 ± 88.39	1134.32 ± 99.47	0.682
Serum Bilirubin, Mean ± SD, mg dL-1		0.72 ± 0.06	0.82 ± 0.05	0.161
Albumin, Mean ± SD, g dL-1		4.44 ± 0.07	4.52 ± 0.06	0.302
Serum creatinine, Mean ± SD, mg dL-1		0.85 ± 0.03	0.81 ± 0.03	0.049
eGFR ^c^, Mean ± SD, ml min-1 1.73 m^2^-1		105.04 ± 4.85	109.08 ± 4.15	0.240
**Entecavir Cohort (n = 20)**				
Age, Mean ± SD, y	44.45 ± 1.94			
Gender, Male (%)	15 (75)			
Cirrhosis, (%)	3 (15)			
HBeAg positive, (%)	15 (75)		15 (75)	
Serum ALT, Mean ± SD, IU L-1	176.70 ± 49.84		31.25 ± 3.72	0.011
Serum AST, Mean ± SD, IU L-1	101.70 ± 22.71		27.95 ± 2.34	0.004
Serum HBV, Mean ± SD, DNA ×10^6^ copies mL-1	379.78 ± 147.60		0.01 ± 0.004	0.030
Serum ACE, Mean ± SD, ng mL-1	190.64 ± 21.27		146.52 ± 13.72	0.020
Serum Bilirubin, Mean ± SD, mg dL-1	0.88 ± 0.14		0.74 ± 0.07	0.489
Serum creatinine, Mean ± SD, mg dL-1	1.06 ± 0.06		1.08 ± 0.08	0.727
eGFR, Mean ± SD, ml min-1 1.73 m^2^-1	80.24 ± 4.19		81.41 ± 5.40	0.805

^a^ Values are presented as mean ± standard error

^b^ Treatment duration: LdT: 330.00 ± 0.85 days; Entecavir: 412.88 ± 36.92 days.

^c^ Abbreviations: ACE, angiotensin converting enzyme; ALT, alanine aminotransferase; AST, aspartate transaminase; CFH, complement factor H; eGFR, estimated glumolular filtration rate; HBV, hepatitis B virus; LdT, Telbivudine.

### 4.3. Serum ACE Was Negatively Correlated with eGFR in the LdT-Treated Cohort

**Figure 2.**
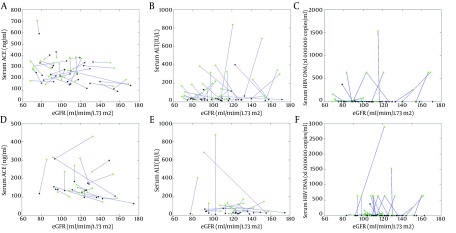
Correlation Between eGFRs and ACE Green and black diamonds represent the values of subjects measured at baseline and after treatment, respectively. (A-C) The LdT cohort. (A) ACE vs. eGFRs (r = -0.375, P = 0.002). (B) ALT vs. eGFR (r = 0.209, P = 0.088). (C) HBV DNA vs. eGFR (r = 0.144, P = 0.241). (D-F) The Entecavir cohort. (D) ACE vs.eGFR (r = -0.239, P = 0.138). (E) ALT vs. eGFR (r = -0.308, P = 0.053). (F) HBV DNA vs. eGFR (r = 0.175, P = 0.299).

A negative correlation was discovered between serum ACE levels and eGFRs in the LdT-treated cohort (r = -0.375, P = 0.002, Figure 2A). The coefficient of determination (R2) was 0.141 (18). ALT did not have a significant correlation with eGFR (r = 0.209, P = 0.088, Figure 2B), neither did serum HBV DNA (r = 0.144, P = 0.241, Figure 2C). At 330.00 ± 0.85 days, serum creatinine levels were reduced with a borderline statistical significance (P = 0.049, Table 3), yet eGFR had not manifested a significant change (P = 0.240, Table 3). A significant increase of eGFR from baseline was found (33 patients, P = 0.002) at 708.64 ± 31.63 days.

No significant correlation between serum ACE levels and eGFRs was found in the entecavir-treated cohort (r = -0.239, P = 0.138; Figure 2D). Serum ALT did not have a significant correlation with eGFR (r = -0.308, P = 0.053; Figure 2E), neither did Serum HBV DNA (r = 0.175, P = 0.299; Figure 2F). No significant change in eGFR was observed (15 patients; P = 0.261) at 1298.33 ± 85.70 days.

## 5. Discussion

The purpose of this exploration study was to find hepatitis-independent, drug-related molecular alterations. ALT is an indication of the severity of hepatitis. By combining ALT-high baseline subjects and ALT-low inactive carriers as the non-treated group, the ALT is not significantly different from the LdT-treated group using the non-parametric Mann-Whitney tests. In this way, we hoped that the hepatitis-dependent genes would not be selected out as candidates. 

This study was a retrospective study, which was limited by the availability of stored, paired or unpaired samples as well as time-dependent clinical data. Serum levels of ACE were not routinely checked previously. Hence, the quantitation of serum ACE levels relied on the stored samples. The use of paired samples before and after the treatment from the same patients can certainly cancel out variability due to individual genetic differences. However, the availability of paired samples is limited. Therefore, in this study, we used paired samples from the same patients only in our verification studies.

We demonstrated a consistent reduction of serum ACE concentration upon two oral antiviral monotherapies, LdT and entecavir. Although eGFR did not show significant improvement at 330.00 ± 0.85 days (P = 0.240), a marked improvement did appear after a longer period of treatment (708.64 ± 31.63 days; P = 0.002), suggesting a possible time-lag effect of eGFR improvement following the drop of serum ACE levels. Samples from patients treated for a longer period (> 2 years) were not available for measuring ACE in the current study. Hopefully, in the future, new projects can be initiated to measure ACE levels in patients undergoing longer periods of treatment.

We also demonstrated a negative correlation between serum ACE concentration and eGFR in the LdT cohort. Data showed that HBV DNA and serum ALT levels, two indicators of hepatitis severity, did not correlate with eGFR. Therefore, hepatitis amelioration cannot explain the improvement of renal function.

Although the suppression of ACE was first found by LdT treatment in the exploratory study, we were intrigued to see whether the effect can or cannot be generalized to other oral antiviral drugs. The ACE levels did not have a significant Pearson’s correlation with eGFR in the entecavir cohort. No significant change in eGFR was found after a longer period of treatment. This could be due to the small sample size of the entecavir group, or alternatively, other unrecognised factors may have hindered renal function improvement among entecavir-treated patients.

The elevation of CFH mRNA expression in the exploratory study offered an independent theory behind renal function improvement, which was not supported by the subsequent verification. CFH was documented to play protective roles against multiple renal diseases (19). Genomic variants of CFH and their receptors were also shown to associate with IgA nephropathy (20). Several other renal function-related genes were also identified in the exploratory phase. However, no reliable standardized ELISAs were available at this time to verify these findings. 

As the prevalence of chronic kidney diseases increased, protection of renal function during antiviral therapy becomes an important issue in the management of chronic hepatitis B patients. Oral antiviral drugs such as LdT may be a reasonable choice for patients whose renal function is of critical concern. For example, LdT may be suitable for the prevention of HBV relapse and kidney damage for advanced hepatocellular carcinoma patients who require renal-toxic chemotherapies. Similarly, LdT can be used to treat HBV patients who also have diabetic nephropathy.

In summary, we discovered significant reductions of serum ACE levels by monotherapies of two oral antiviral drugs, LdT and entecavir. The eGFRs and ACE levels were negatively correlated in LdT-treated patients, and eGFR significantly increased after long-term LdT treatment. This finding partly explained the apparent renal function improvement in chronic hepatitis B patients receiving LdT therapy.
